# CSACI position statement: safety of topical calcineurin inhibitors in the management of atopic dermatitis in children and adults

**DOI:** 10.1186/1710-1492-9-24

**Published:** 2013-07-09

**Authors:** Audrey O Segal, Anne K Ellis, Harold L Kim

**Affiliations:** 1 Department of Paediatrics, Rouge Valley Health System, Toronto, ON, Canada; 2 Division of Allergy & Immunology, Department of Medicine, Queen’s University, Kingston, ON, Canada; 3 Department of Biomedical & Molecular Sciences, Queen’s University, Kingston, ON, Canada; 4 Division of Allergy & Immunology, Department of Medicine, University of Western Ontario, London, ON, Canada; 5 Division of Allergy & Immunology, Department of Medicine, McMaster University, Hamilton, ON, Canada

**Keywords:** Atopic dermatitis, Topical corticosteroids, Therapy, Tacrolimus, Pimecrolimus, Lymphoma, Drug safety

## Abstract

Atopic dermatitis (AD) is a condition frequently encountered in medical practices across the country. Arming ourselves with appropriate and safe treatment modalities to provide relief for this chronic and relapsing inflammatory condition is of utmost importance to our patients and their families. Utilizing topical calcineurin inhibitors (TCIs) for the treatment of AD not responsive to high-potency corticosteroids, or low-potency corticosteroids and localized to the face, eyelids, and skin folds of patients >2 years, is reasonable to include in common practice. Despite the FDA’s Black Box warning, to date no evidence has been published linking the TCIs to an increased incidence of malignancy in either children or adults that establishes causation. The Canadian Society of Allergy and Clinical Immunology (CSACI) therefore recognizes that the benefits of TCIs should be carefully weighed with the theoretical risks in advising patients, and acknowledges that long-term studies remain in progress. The safety and efficacy of topical tacrolimus and pimecrolimus should therefore be considered when treating children and adults with AD in Canadian allergy and immunology practices.

##  

Atopic dermatitis (AD), a chronic and relapsing inflammatory skin condition, is often the first manifestation of atopy in children [1,2]. Up to 71.3% of children with AD report allergic rhinitis and/or asthma [3]. Managing AD is therefore of utmost importance to the practicing clinical immunologist and allergist.

The hallmarks of AD include pruritus and ill-defined erythema, with edema and/or vesicles if acute, and lichenification if chronic. It has a predilection for the flexural creases, or, in the case of children under 4 years of age, the cheeks, forehead and extensor surfaces of the limbs [4]. AD is characterized by both structural abnormalities and immune dysregulation, with evidence of both innate and adaptive dysfunction [5]. Effective management of AD involves measures to avoid exacerbating triggers, maintenance of skin barrier function and therefore hydration, management of pruritus, and treatment of inflammation with anti-inflammatory medications.

Low to intermediate-potency topical corticosteroids have long been utilized as first-line therapy for AD not adequately managed by emollients alone. However, potent fluorinated corticosteroids are not indicated for use on the face, eyelids, genitalia, intertriginous areas, or in young infants. Topical calcineurin inhibitors (TCIs), such as tacrolimus and pimecrolimus, are alternatives to corticosteroids for AD on the face, eyelids and skin folds that is unresponsive to low potency topical steroids [6].

TCIs exert their anti-inflammatory action by inhibiting calcineurin-dependant T-cell activation, thus inhibiting the activation of pro-inflammatory cytokines and mediators of the allergic inflammatory reaction [7]. They have also been demonstrated to exert effects on mast cell activation [8], resulting in relief of pruritus and erythema as early as within three days of initiating treatment [9]. Topical tacrolimus has also been shown to decrease both the number and co-stimulatory ability of epidermal dendritic cells [10]. However, it is these anti-inflammatory mechanisms of action which have caused concern that high or prolonged use may lead to malignancy much like those related to oral use of these immunosuppressives.

A recent review article by Frankel et al. concluded that tacrolimus 0.1% ointment has been demonstrated to be as effective as mid to high potency class III-V steroids in the treatment of AD [10]. Both tacrolimus and pimecrolimus have been associated with an increased frequency of application-site reactions (namely, localized transient burning), but also with a decreased risk of skin atrophy as compared to topical corticosteroids [10].

Despite evidence demonstrating efficacy, resistance to using the TCIs in management of AD is prevalent among clinicians. This anxiety arises in part from the release of an FDA Black Box warning in 2005, citing concerns that chronic intermittent use of TCIs could lead to an increased incidence of Hodgkin’s and non-Hodgkin’s lymphoma, as well as melanoma and non-melanoma skin cancers [11]. Following the release of this warning, many physician groups, including the American Academy of Allergy, Asthma and Immunology (AAAAI), American College of Allergy, Asthma and Immunology (ACAAI), and the Canadian Dermatology Association (CDA), released position statements, promoting the safety of the TCIs, and arguing against the FDA warning [12-14]. In an effort to promote appropriate use of the TCIs in Canadian clinical practice, the CSACI has developed this position statement.

Importantly, the FDA warning was one of a *theoretical* risk of increased malignancy with the use of TCIs. This was based primarily on animal data, case reports and the mechanism of action of the drugs (where tacrolimus, *in vitro*, has been demonstrated to inhibit spontaneous DNA repair [15]). Of note, however, there has been no strong evidence published that this could represent a similar risk in humans. In fact, in 2005, the spontaneous reporting system described fewer cases of malignancy in patients treated with TCIs than would be expected in the population over the same period of time [13]. More recent data continue to show similar incidence rates [16]. To date, no evidence has been published that concludes a causal relationship between malignancy in patients with AD, and the use of TCIs [13,14,17]. This includes several nested case–control studies demonstrating no increased risk of lymphoma in AD patients being treated with TCIs, compared to those without TCI exposure [18,19]. Preliminary data from long-term safety studies have demonstrated similar safety profiles, though these studies are ongoing [20]. A recent retrospective cohort observational study did show a possible risk of increased incidence of T-cell lymphoma in patients with AD treated with TCIs, however, this was possibly attributed to a bias in its use early in this condition. There was no increase in other malignancies demonstrated in this study [21]. Notably, the alternatives for patients with AD refractory to low-moderate potency corticosteroids (i.e. switching to high potency topical steroids and/or leaving AD untreated) carry an even higher risk profile and/or will lead to ongoing patient suffering. It is important to note, however, that younger subjects with a higher body surface area per weight, and subjects with abnormal epidermis (ie. Netherton’s syndrome), can have significant percutaneous absorption of topically applied calcineurin inhibitors, which may result in systemic serum concentrations known to cause immunosuppression. This is why these drugs are not indicated for use in children under 2 years of age, or patients with severely impaired skin barrier function (ie. patients with Netherton’s syndrome).

In summary, topical calcineurin inhibitors are effective treatments for atopic dermatitis, and the benefits of their use in the appropriately selected patient population outweighs the theoretical risk of increased malignancy.

## Key Points

1. Low- to intermediate-potency topical corticosteroids are first-line therapy for AD. However, intermediate-potency topical corticosteroids are not indicated for long-term use on the face, eyelids, genitalia, and intertriginous areas.

2. Topical calcineurin inhibitors (TCIs) are indicated for AD in patients 2 years of age and older.

3. There is no current published evidence showing that TCIs clearly predispose to malignancy.

## Abbreviations

CSACI: Canadian Society of Allergy and Clinical Immunology; AD: atopic dermatitis; TCI: topical calcineurin inhibitor; FDA: Food and Drug Administration; AAAAI: American Academy of Allergy, Asthma and Immunology; ACAAI: American College of Allergy, Asthma and Immunology; CDA: Canadian Dermatology Association.

## Competing interests

This position statement did not receive financial support from any industry sources.

Dr. Audrey O. Segal has served on an Advisory board for Sanofi. Dr. Anne K. Ellis has served on the speaker’s bureau for Merck, Pfizer and Sanofi, an Advisory board for Paladin Labs and Sanofi, and has received research grants from Adiga Life Sciences/Circassia Ltd. Dr. Harold L. Kim has served on the speaker’s bureau for Astellas, AstraZeneca, Merck, Novartis, Pfizer and Takeda, and on Advisory boards for CSL Behring, Merck and Novartis.

## Authors’ contribution

This Position Statement was the product of an ad hoc committee of the Canadian Society of Allergy and Clinical Immunology. Each of the credited authors contributed substantially throughout the planning, drafting and revision stages of the document, and all authors read and approved the final manuscript.
